# Sensor-Less and Control-Less Underactuated Grippers With Pull-In Mechanisms for Grasping Various Objects

**DOI:** 10.3389/frobt.2021.631242

**Published:** 2021-02-22

**Authors:** Atsushi Kakogawa, Yuki Kaizu, Shugen Ma

**Affiliations:** Department of Robotics, Ritsumeikan University, Shiga, Japan

**Keywords:** underactuation, robotic gripper, sensor-less, differential mechanism, pneumatic gripper, in-hand manipulation

## Abstract

This paper proposes an underactuated grippers mechanism that grasps and pulls in different types of objects. These two movements are generated by only a single actuator while two independent actuators are used in conventional grippers. To demonstrate this principle, we have developed two kinds of gripper by different driving systems: one is driven by a DC motor with planetary gear reducers and another is driven by pneumatic actuators with branch tubes as a differential. Each pulling-in mechanism in the former one and the latter one is achieved by a belt-driven finger surface and a linear slider with an air cylinder, respectively. The motor-driven gripper with planetary gear reducers can pull-up the object after grasping. However, the object tends to fall when placing because it opens the finger before pushing out the object during the reversed movement. In addition, the closing speed and the picking-up speed of the fingers are slow due to the high reduction gear. To solve these drawbacks, a new pneumatic gripper by combining three valves, a speed control valve, a relief valve, and non-return valves, is proposed. The proposed pneumatic gripper is superior in the sense that it can perform pulling-up after grasping the object and opening the fingers after pushing-out the object. In the present paper, a design methodology of the different underactuated grippers that can not only grasp but also pull up objects is discussed. Then, to examine the performance of the grippers, experiments were conducted using various objects with different rigidity, shapes, size, and mass, which may be potentially available in real applications.

## 1 Introduction

End-effectors have been widely used in the industry to grab, place, and handle various objects. In particular, human-like robotic hands have potential for dexterous and versatile tasks. However, this requires more actuators, redundancy, and complicated control systems, which leads increased cost, delivery time, and maintenance difficulty. In this context, one of the key challenges is to simplify the manufacture of the end-effector without losing performance. To address this issue, many robotic grippers with fewer fingers specially designed for particular tasks have been investigated.

Nagata (1994) proposed a gripper with two turntables that can rotate objects. This device can hold and tighten or loosen a screw by alternating the rotational direction of each turntable. Kabaya and Kakikura (1998) developed a gripper with two active rollers that can grasp and pull-in a sheet-like object on a flat ground. Yuan et al., (2020) also proposed a gripper with two active rollers. Combined with a linkage mechanism, it can change the rollers’ orientation for within-hand manipulation. Tadakuma et al., (2012) developed a finger mechanism equipped with an omnidirectional driving roller with two active rotational axes that can grasp and pull-in objects. A particular aspect is that such devices allows rotation of the object around any axis and translation of the object along the fingers. Tincani et al., (2012) and Krug et al., (2014) created a finger mechanism with two degrees of freedom and an active surface that can tightly grasp the object by pulling-in it, even when the object is pinched with the fingertips. The fingers and additional gripping mechanisms are independently controlled using many actuators and sensors.

Compared to the sophisticated human-like robotic hand, grippers with multi-active degree of freedom can reduce the number of actuators. However, there is still room to reduce them further by using differential mechanism. Underactuated grippers with differential mechanisms and fewer sensors and actuators have been reported to simplify control. Hirose and Umetani (1978) presented a soft gripper composed of a novel mechanism called “articulated differential” that can passively adapt to the shape of the object during the grasp. This theory has been applied to various fingers, such as a differential gear chain by Tamamoto and Koganezawa (2013). In this context, underactuated grippers with various types of differential mechanisms (e.g., pulley, gear, linkage, and bar) have also been investigated by Laliberte and Gosselin, (1998), Gosselin and Laliberté (2010), Ozawa et al., (2016), Wang et al., (2002).

However, in most underactuated grippers, the differential mechanism was installed between each output of the joints to grasp while fitting the finger to the object shape. The movements of two outputs are essentially equal (both are joint rotation on the common plane). Our idea lies in the conception of underactuated grippers with two different movements: finger-closing and pulling-in the object. There are two main reasons why the pulling-in movement is valid as an end-effector. First, when the gripper with a pull-in mechanism is attached to robotic manipulators, the end robot does not have to be moved up to grab the object up. The gripper itself can move the object up from the ground. Second, the object can be stably supported by three points (surfaces of the two fingers and base) after being pulled in.

In addition, the combination of this pull-in mechanism and differential mechanism is further valid for many applications. For example, it does not require feedback control and tactile sensors for the switch of the two movements. Since the robotic finger of the proposed gripper is composed only of simple mechanical parts, the gripper is waterproof and can be therefore submerged into the water without any damage. Even if the fingers get dirty, the gripper can be cleaned with water. When one output movement shifts to another, the gripping force can be adjusted by regulating the resistance of one side of the outputs. Due to this passive mechanism, the device can grip fragile objects without smashing.

**FIGURE 1:**
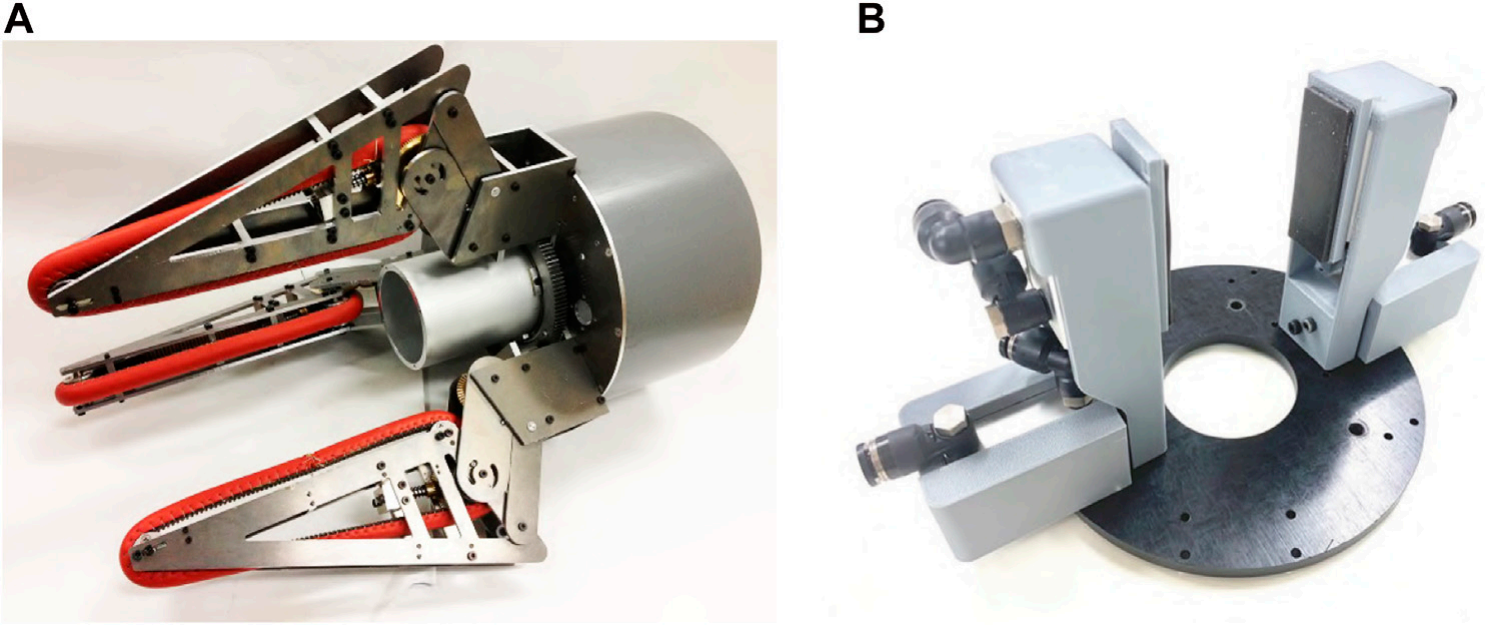
The motor-driven **(A)** and pneumatic **(B)** grippers with underactuated pull-in mechanisms.

Previously, we developed a three-fingered underactuated gripper that can grip and pull-in objects (Nishimura et al., 2012; Kakogawa et al., 2016) as shown in Figure 1A. An electric geared motor and a planetary gear reducer were used in both to produce two types of movement. However, there were still many challenges to be solved, such as speed, weight, and size. Therefore, to solve the problems of our previous underactuated grippers with pull-in mechanisms, we propose a new type of gripper with pneumatic actuators and pneumatic differential mechanism (Figure 1B; Table 1). In this paper, our previous works are briefly explained, followed by our new gripper.

**TABLE 1:** Specification difference between the previous motor-driven gripper and the proposed pneumatic gripper.

Parameter	Motor-driven	Pneumatic
Whole weight [kg]	4.8	0.57
Weight of a single finger unit [kg]	0.9	0.21
Whole size [mm]	381 × 381 × 495	195 × 140 × 110
Theoretical maximum gripping force (a single finger) [kgf]	41.2	11.5
Theoretical maximum payload (a single finger) [kgf]	62.6	11.5
Theoretical maximum gripping speed (a single finger) [m/s]	0.01	0.5
Theoretical maximum pulling-in speed (a single finger) [m/s]	0.015	0.5

## 2 Underactuated Grippers With PULL-IN Mechanisms

**FIGURE 2:**
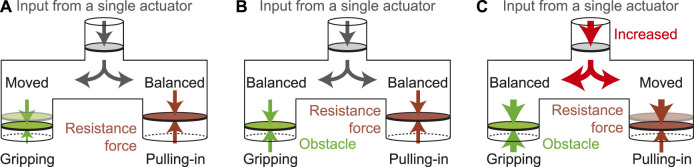
Branch tube models for the principle of the proposed underactuated gripper: **(A)** Until gripping an object, **(B)** Once gripping an object, **(C)** Pulling-in an object after gripping.

Figure 2 presents the branch tube models to explain the differential actuation concept of our proposed underactuated grippers. The principle of underactuation is identical for both motor-driven and pneumatic types. The input from the actuator is applied to both the outputs. Therefore, both of them move simultaneously when the input is applied. However, only one side of the outputs moves if another side is restricted by external force and the input is continuously applied. Generally, a spring or friction with a mechanical contact has been used to obtain this external force. In our case, the branched input force is balanced with the external force at the pulling-in side; this force is applied based on the resistance regulation mechanism (motor-driven gripper) or the relief valve (pneumatic gripper). Therefore, only the gripping side moves when the input is provided at first. Once the fingers grip the object completely, their movement is restricted. Thus, the input force increases, which corresponds to an increase in motor current. Because the input is connected to both the outputs through the differential mechanism, both the forces for gripping and pulling in increase. Then, the pulling-in force exceeds the initially applied resistance. Thus, the object can be pulled in within the gripper. Our proposed grippers work based on this principle regardless of the actuator type.

### 2.1 Previous Work: Motor-Driven Gripper

**FIGURE 3:**
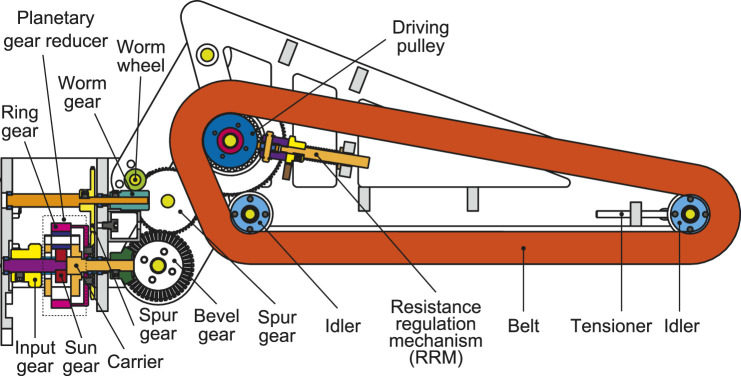
Cross-sectional view of an underactuated modular finger.

A cross-sectional view of our previous gripper with an electric motor and planetary gear reducer is shown in Figure 3. It comprises a parallel link mechanism to keep the pull-in belt in constant alignment. The belt itself is made from a silicone rubber sponge and has a semicircular cross-section for grasping objects of various shapes.

An input gear connected to a motor drives the Sun gear of the planetary gear reducer. Because the carrier and the ring gear of the planetary gear reducer are not fixed, the torque from the motor is distributed to two outputs. The carrier output is transmitted to the pull-in belt through the gear chain mechanisms, while the ring-gear output is used for gripping the object. When the gripper is directed vertically downward, the fingers tend to close on their own because of gravity. To stop this movement, a worm gear without back-drivability was used.

**FIGURE 4:**
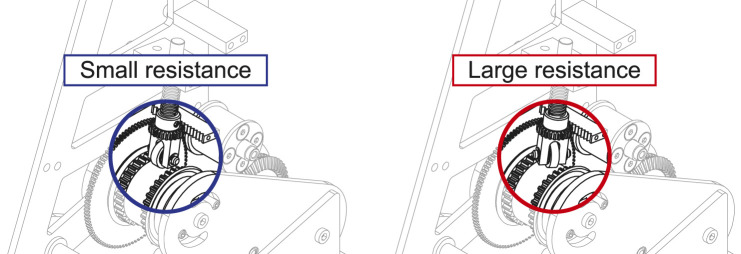
Resistance regulation mechanism (RRM).

Considering the picking movement, the fingers should grip the object first before pulling-in. However, to know which of the two outputs should come first depends on how much resistance is applied to each finger and pull-in belt. Therefore, a resistance regulation mechanism (RRM) is designed for the driving pulley, as shown in Figure 4. A coil spring that presses a free rubber roller onto the driving pulley is installed. The angle of the roller can be manually set from 0° to 90° by a rack and pinion. The resistance is minimal when the angle is 0° because the roller turns freely with the pulley. However, the frictional resistance increases with the angle to which the roller is set, reaching a maximum at 90°. The torque distribution of the differential mechanism can be tuned by setting this angle beforehand.

In our previous work, it was revealed that the motor-driven underactuated gripper with pull-in mechanism can pull in the object after gripping without sensor and control. However, the following disadvantages were found. First, the gripping speed is considerably reduced due to the worm gear at the gripping side to avoid backdrive. Second, to distribute a single input to two outputs (gripping and pulling-in) through a differential mechanism (planetary gear reducer), complicated gear chain mechanisms are necessary. This leads to the increase in size and weight of the entire gripper. Third, friction force was used for the resistance force to avoid the movement of two outputs at the same time. In the motor-driven underactuated gripper, a free rubber roller was installed. However, the friction condition is difficult to pre-estimate and the attrition of the rubber is not negligible. Forth, the fingers open ahead of pushing-out of the object when reversing the motor’s rotational direction; this is the most critical problem. As a result, the object drops down, which may cause breakage.

In the present paper, to solve the above problems, a pneumatic actuator was adopted. Because the source of the power of the pneumatic actuator is air, differential mechanism can be easily achieved by using a simple branch tube. The resistance force applied to the differential mechanism can be given by various valves that have been already standardized for industrial use. Furthermore, the problem of the motion sequence when releasing can be solved by combining different valves. The principle of the proposed pneumatic gripper is explained in more detail from the next sections.

### 2.2 New Gripper: Pneumatic Gripper With Various Valves and Branch Tubes

**FIGURE 5:**
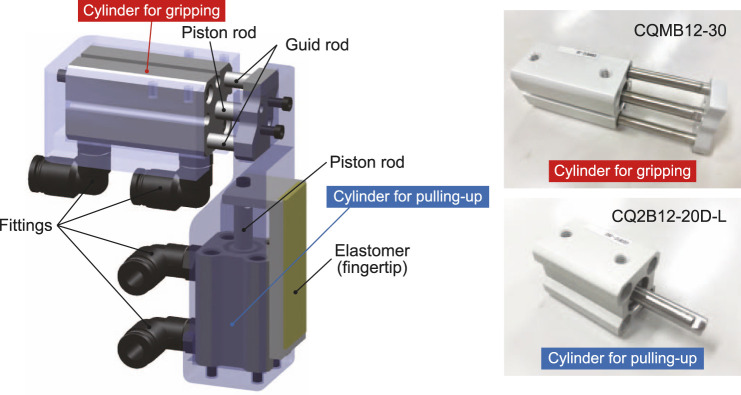
A single finger unit of the proposed pneumatic underactuated gripper.

Figure 5 depicts a single finger unit of our proposed pneumatic gripper. It comprises two air cylinders: one is for gripping and opening (CQMB12-30, manufactured in SMC Corporation, Tokyo, Japan) and another is pulling-in and pushing-out objects (CQ2B12-20D-L, manufactured in SMC Corporation, Tokyo, Japan). These movements are shifted automatically using a differential air circuit (simple branch tube) without the need for a sensor or microcontroller.

Operators only change the air flow direction using directional control valves when picking (gripping and pulling in) and placing (pushing out and finger opening). No other operation is needed; thus, within-gripper pick and place are easily possible. Each cylinder has two fittings (air entrance and exit) for extension and compression. When the air flow direction is switched, the entrance and exit of the cylinder fittings are swapped. To increase the frictional coefficient between the finger and the object, a silicon elastomer is attached to the fingertip. Because it is soft material, the fingertip surface can change its shape to fit the object.

**FIGURE 6:**
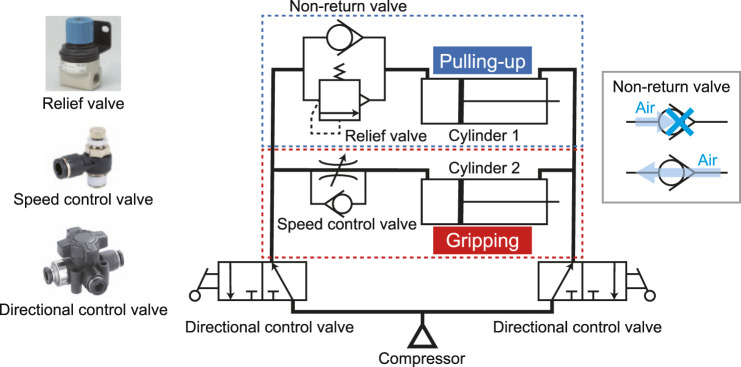
Air circuit of the proposed pneumatic underactuated gripper (one single unit).

Figure 6 illustrates the air circuit of the pneumatic gripper. It has two directional control valves, a speed control valve on the cylinder for gripping, and a relief valve and check valve on the cylinder for pulling up. The speed control valve involves a check valve and throttle valve in parallel in which the air speed can be tuned. The air flow from the opposite side of the speed control valve can pass through the check valve. The relief valve is used to avoid damage of the pipe rupture owing to excessive pressure.

Normally, the valve is closed by the elastic force of the spring. However, the valve opens when the pressure inside the pipe rises. The two movements between gripping and pulling-in are switched using the speed control valve and the relief valve. Because the relief valve cannot allow the reverse air flow, the check valve was installed. Two directional control valves change the direction in which air is sent and the direction in which air is released. This enables gripping and releasing objects with one compressor.

**FIGURE 7:**
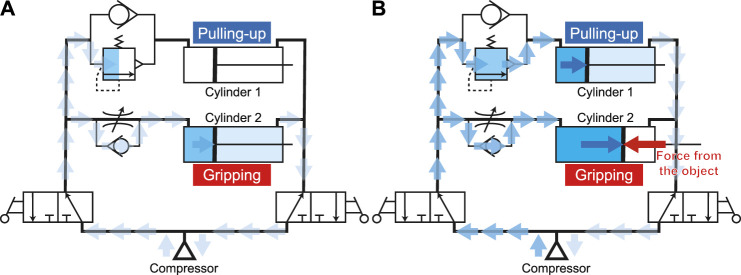
Air flow during **(A)** gripping process and **(B)** pulling-in process.

First, the air flows from the compressor to the left side of the circuit as shown in Figure 7A. In this state, the right side of the circuit is atmospheric pressure. Because the relief valve is closed, the compressed air does not flow to the cylinder for pulling-in and the cylinder for gripping only moves. When two fingers contact and sandwich an object, the cylinder for gripping cannot move any further (the gripping force and reaction force from the object are balanced); thus, it stops. Then, the internal pressure of the tube in the circuit rises.

When the pressure in the circuit exceeds the limitation set by the relief valve, the valve opens and the cylinder on the pull-up side is driven (Figure 7B). When the pulling-in operation is finished, the cylinders are full of the compressed air, and the supply from the compressor stops. Since this gripper is driven by air pressure, it is possible to maintain the gripping force as long as the inner pressure of the cylinder does not fall.

**FIGURE 8:**
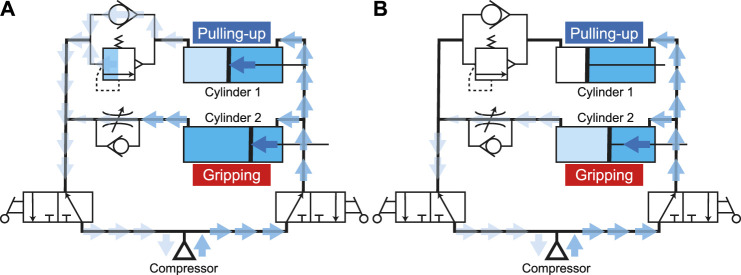
Air flow during **(A)** pushing-out process and **(B)** opening process.

After the gripping and pulling-in movements, the air remains in the circuit and cylinders. When the air supply direction from the compressor is changed by two directional control valves, the air is supplied to the right side of the circuit and the air on the left side escapes to the atmosphere, as shown in Figure 8A. It is difficult for the air in the cylinder for gripping to decrease because of the speed control valve. Therefore, it is difficult to open the fingers because of the residual pressure remaining in the cylinder. On the other hand, since the cylinder for pulling-in is easy to move, the previously pulled object is pushed-down to the tip. Finally, the air remaining in the cylinder for gripping gradually decreases, while the resistance due to the residual pressure also decreases. Then, the cylinder starts moving and the two fingers are opened (Figure 8B).

## 3 Experimental Verification

### 3.1 Experiments to Verify the Performance of the Proposed Pneumatic Differential Mechanism

To verify whether the proposed underactuated pneumatic gripper works, the inner air pressure of the tube was measured. The validity of the relief valve and the speed control valve was also examined. The pressure sensor, DP-102ZA-J (Panasonic Corporation, Osaka, Japan) was installed between the cylinder for gripping and the speed control valve. The distance between the object and the base disk of the gripper was also measured using GP2Y0E02A (Sharp Corporation, Osaka, Japan). A POM (polyoxymethylene) block (120 mm × 50 mm × 30 mm in size and 0.2715 kg in weight) was used as an object.

**FIGURE 9:**
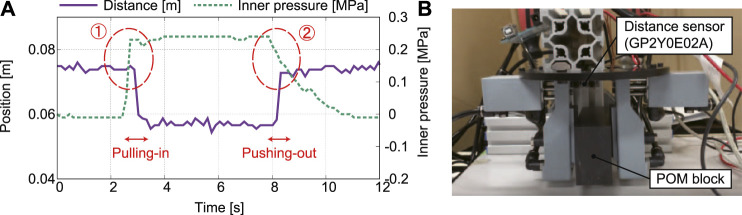
Results of the preliminary experiments: **(A)** measured distance and inner pressure and **(B)** experimental setup.

Figure 9 shows the measured results of the distance and pressure sensor and the experimental setup. Since the distance between the object and the base disc of the gripper obviously changed as plotted in Figure 9A, it is clear that the pulling-in and pushing-out movements of the object were both succeeded. At point ①, the internal pressure of the cylinder increased sufficiently before the output was switched. This implies that two fingers of the gripper close and then contact the object once the air is supplied from the compressor. Subsequently, the movement of two fingers is constrained, leading to increased inner pressure (the gripping force increases). Then, the relief valve opens and the pull-in movement starts. This can be also clarified from the fact that the internal pressure drops momentarily when the output is switched. This phenomenon occurred because the relief valve opened and the volume in the circuit suddenly increased. Focusing on ②, it can be observed that the internal pressure of the gripping cylinder remains when the object returns to its original position. Therefore, the object was first pushed-down to the fingertips, and then two fingers opened. However, at the moment that the pushing-out movement completed, the internal pressure of approximately 0.08 MPa decreased. After that, it gradually decreased to 0 MPa.

**FIGURE 10:**
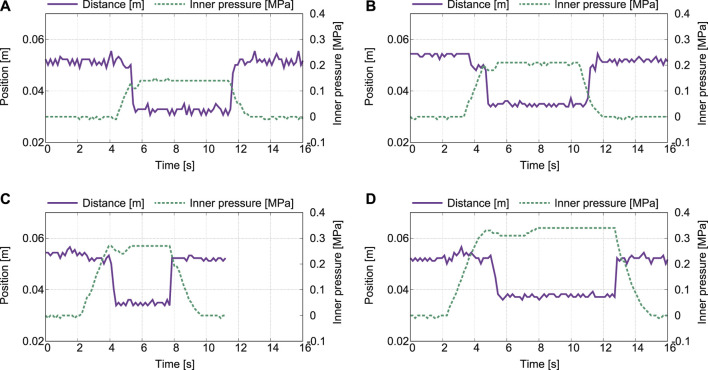
Measured distance and pressure with different throttle amount of the relief valve: **(A)** 2.5, **(B)** 3.0, **(C)** 3.5, and **(D)** 4.0

To test the effectiveness of the relief valve while changing its throttle amount in four stages, this study verified how the magnitude of the internal pressure in the cylinder changes. The input pressure from the compressor was set to 0.4 MPa. Figure 10 plots the measured distance and inner pressure with different throttle amounts of the relief valve: from (a) 2.5 to (d) 4.0 with the interval of 0.5. First, it can be observed that the object can be gripped and pulled in, pushed out, and released in all experiments. Regarding the moment of switching the outputs, the measured internal pressures was 0.13 MPa when the throttle amount was 2.5, 0.2 MPa when the throttle amount was 3.0, 0.27 MPa when the throttle amount was 3.5, and 0.33 MPa when the throttle amount was 4.0. It was confirmed that the internal pressure of the cylinder increases when switching the outputs as the amount of throttle increases.

### 3.2 Experiments to Verify the Performance of the Proposed Underactuated Pneumatic Gripper With Different Objects

To verify the performance of the proposed underactuated pneumatic gripper, whether it can grip, pull-in-in, push-out, and release objects of different shapes or not was examined (see Supplementary Material). The different objects are listed in Table 2. A POM block and a pen are very ordinal objects; thus, it is generally easy to grip with a robotic gripper. Only difference between them is the surface shape (flat and arc). However, it is difficult to pick thin objects up because the fingertip touches the ground that disturbs its closing movement. To test this hard task, a thin metal plate and a towel placed on the flat ground are prepared.

**TABLE 2:** The size and weight of the objects.

Object	Size [mm]	Weight [kg]
POM block	120 × 50 × 30	0.272
Pen	ϕ11 × 148	0.027
Metal plate	42 × 42 × 2	0.009
Towel	29 × 20 × 3	0.018

**FIGURE 11:**
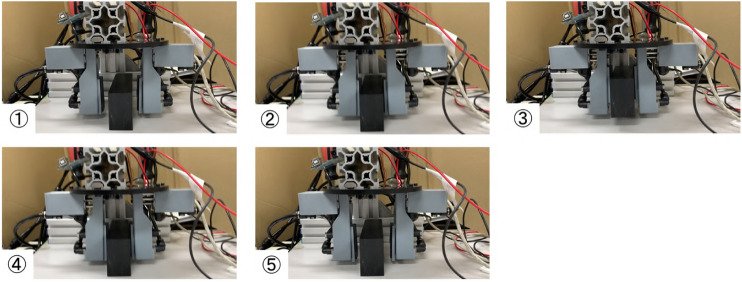
Gripping and releasing a POM block with two fingered pneumatic gripper.

**FIGURE 12:**
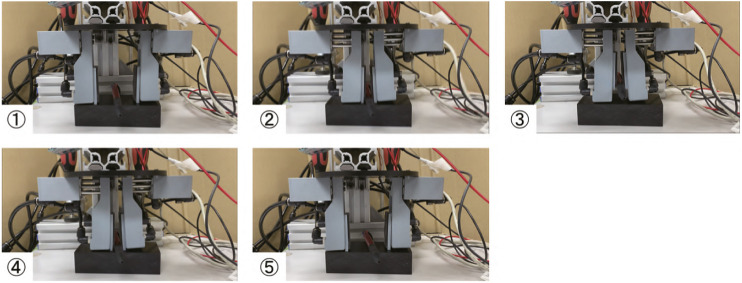
Gripping and releasing a pen with two fingered pneumatic gripper.

**FIGURE 13:**
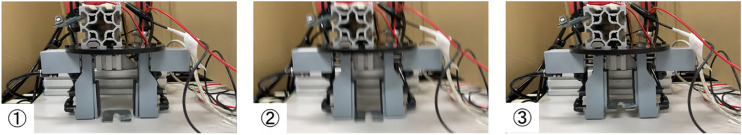
Gripping and releasing a metal plate the two fingered pneumatic gripper.

**FIGURE 14:**
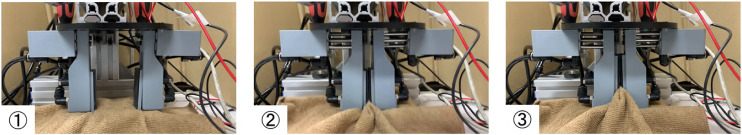
Gripping and releasing a towel with two fingered pneumatic gripper.

Figures 11–14 show the video cut-out of the experiments. Experimental results of grasping and pulling-in movement and that of pushing-out and finger-opening movement are listed in Table 3. It was confirmed that the proposed pneumatic gripper can grip, pull-in-in, push-out, and release all objects with the expected sequence. However, the pen fell when released because two fingers did not push-out at the same time. Similar phenomena happened when releasing the metal plates. The metal plate fell more often than the pen even if the tilt angle is small.

**TABLE 3:** Experimental results of gripping, pulling-in, pushing-out and finger-opening with different objects.

	Grip and pull-in					Push-out and finger-open				
**Object**	**1st**	**2nd**	**3rd**	**4th**	**5th**	**1st**	**2nd**	**3rd**	**4th**	**5th**
POM block	○	○	○	○	○	○	○	○	○	○
Pen	○	○	○	○	○	○	×	×	○	○
Metal plate	○	○	○	○	○	×	×	○	×	×
Towel	○	○	○	○	○	△	△	△	△	△

○ *:* success, × *:* failure, and △ *:* conditional success.

Since the fingertip is supported by only one side of the piston rod, the rod slightly tilts when the object cross-sectional area is small and the contact point is close to the fingertip. As a result, the frictional condition between the rod and cylinder case in the right and left fingers could change. Therefore, an additional resistance force to disturb the pushing-out movement is applied. The possible solution for this problem could be the support of the fingertip by both side of the piston rod. However, additional slider mechanism is necessary that leads to an increase in size and weight.

On the other hands, the towel could be gripped and pulled -in by pressing the gripper against it. However, two fingers were difficult to open after pushing-out-out due to the friction between the towel and the ground. If the object is very thin like a towel, the fingertip may contact with the ground in the pushing-out process. This problem never happens if it is placed on a small stand or table that the width is smaller than that of the object. However, since the gripper grips the towel while folding and bending, it is compressed. Thus, the distance between the fingers is tiny after gripping. This could be solved by regulating the throttle amount of the relief valve. If the gripping force when the movement is switched is small, the gripper can grip the object without shortening the distance between the fingers.

### 3.3 Experiments to Verify the Performance of the Proposed Underactuated Pneumatic Gripper With Heavier Objects

The experiments mentioned above verify the performance of the gripper with light objects. To test the performance with heavier objects more than 0.5 kg in weight, experiments with different PET bottles are conducted. Pick and place of the PET bottles is a good example because it has been widely implemented in factory automations.

**FIGURE 15:**
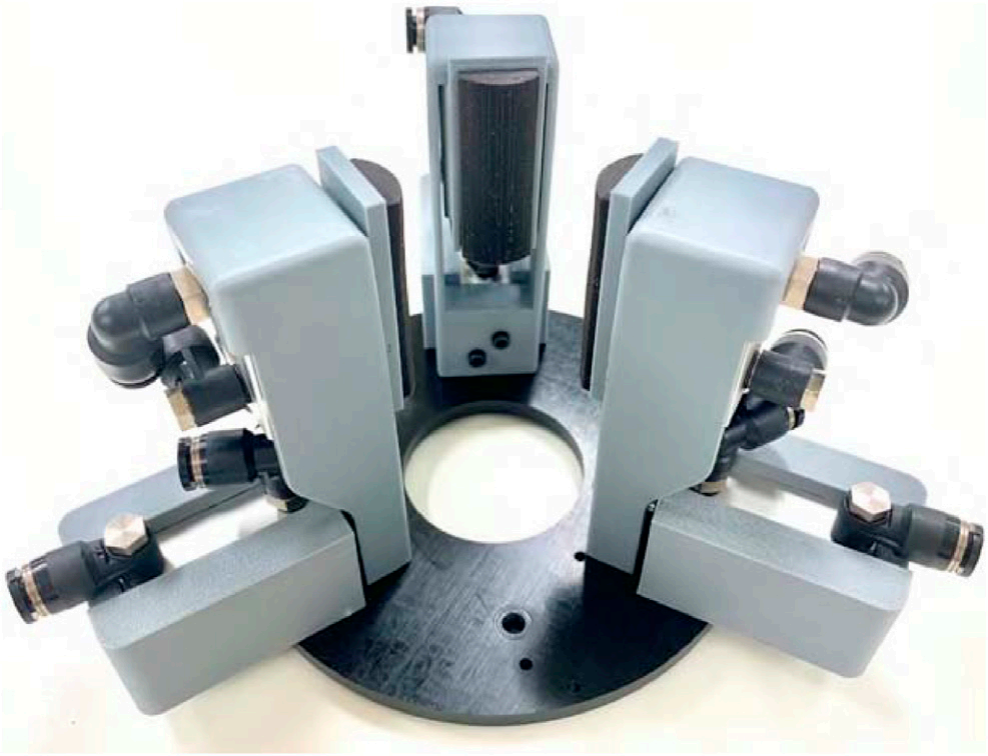
Three-fingered pneumatic gripper with pull-in mechanisms.

Figure 15 depicts three-fingered pneumatic gripper with pull-in mechanisms. Each finger unit is arranged in a circle with point symmetry. Three PET bottles are prepared as listed in Table 4. With only two fingered units, it cannot pull-in and move up 2 L PET bottle (0.525 L and 0.9 L bottle can be pulled-in). Therefore, in this experiment, the performance of three-fingered gripper is examined.

**TABLE 4:** The size and weight of the PET bottles.

Object	Cap size [mm]	Weight [kg]
0.525 L bottle	ϕ30 × 15 × 30	0.547
0.9 L bottle	ϕ30 × 15	0.935
2 L bottle	ϕ30 × 15	2.054

**FIGURE 16:**
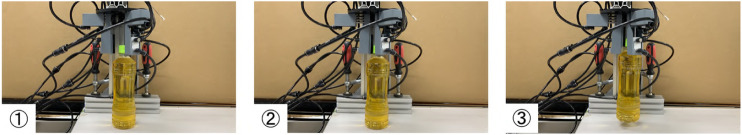
Gripping and releasing a 0.525 L PET bottle with three-fingered pneumatic gripper.

**FIGURE 17:**
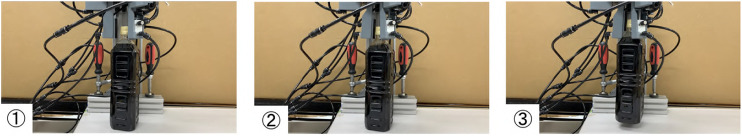
Gripping and releasing a 0.9 L PET bottle with three-fingered pneumatic gripper.

**FIGURE 18:**
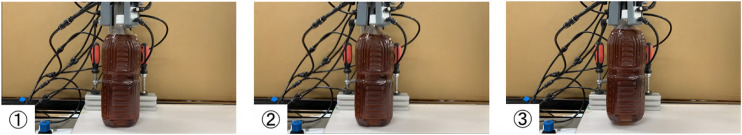
Gripping and releasing a 2 L PET bottle with three-fingered pneumatic gripper.

Figures 16–18 show the video cut-out of the experiments with PET bottles. Their experimental results of grasping and pulling-in movement and that of pushing-out and finger-opening movement are listed in Table 5. From the experimental results, it was confirmed that the proposed three-fingered pneumatic gripper can grip and pull-in PEA bottles filled with liquid although it sometimes slipped and fell when 2 L (20%).

**TABLE 5:** Experimental results of pushing-out and finger-opening movement with different PET bottles.

	Grip and pull-in					Push-out and finger-open				
Object	1st	2nd	3rd	4th	5th	1st	2nd	3rd	4th	5th
0.525 L bottle	○	○	○	○	○	○	○	○	○	○
0.9 L bottle	○	○	○	○	○	○	○	○	○	○
2 L bottle	△	○	○	○	○	×	○	○	○	○

○ *:* success, × *:* failure, and △ *:* conditional success.

## 4 Conclusion

This study proposes sensor-less and control-less underactuated grippers with pull-in mechanisms. First, our previously developed motor-driven gripper was introduced, and its disadvantages were discussed. Next, a newly developed pneumatic gripper that solves the problems of speed, size, weight, and reversed movement (push-out and finger-opening) was explained. Despite the remarkably simple finger structure, the gripper can grip, pull-in, push-out, and release various types of objects. Placing the differential (branch tube) and resistance mechanisms (relief valve and speed control valve) outside of gripper is one of the main reasons why our proposed gripper made our objective possible.

Despite our valuable result, there is scope for further investigation. In the release movement, when there is almost no time difference between the moment that the push-out is completed and the moment that the finger opens, the object may hit the ground and suffer impact. If the object is fragile and deformable, this can lead to its breakage. Therefore, it is necessary to further consider the throttle amount of the speed control valve for the soft releasing movement.

When gripped with the fingertips, two cylinders for the push-out were deviated. Accordingly, the object was rotated slightly. This is due to the low stiffness of the finger body parts. In this research, it was created with a 3D printer to reduce the weight. Thus, the fingertips open compared to the finger base when gripping the object with large force. This causes the resistance to pull-in the object is influenced. How to design the rigid and lightweight finger unit is thing to do next.

The proposed pneumatic gripper was small, lightweight, control-less, and driven by a single compressor. However, the more the number of the finger increases, the more the number of cylinders increases due to modularization. As cylinders increases, it is difficult to synchronize the movement of the multiple cylinders without control. New mechanical design and structure idea are necessary to reduce the number of cylinders for further weight reduction. Sliders for the gripping movement could be shared in only a single cylinder. We are investigating the above problems and will improve the gripper in the future.

Another thing to do is design optimization of the gripper. The most important parameter is the reduction ratio of the underactuated mechanism (differential mechanism). In the present paper, the reduction ratio corresponds to the cross-sectional area of the cylinder. Currently, this area is set to identical in both cylinders for gripping and pulling-in. However, if the cross-sectional area in one of the two outputs is designed greater than that of another, the pneumatic force of the cylinder increases although the speed decreases (note that it is assumed that the inner pressure is constant). The underactuated movement can be generated as normal.

In our case, for example, the time until the gripping cylinder is filled with the air becomes longer and the gripping force increases if its cross-sectional area is designed larger than that of the pulling-in cylinder. This can also take advantage for the reverse movement. Since the time until the gripping (finger-opening) cylinder is filled with the air is extended, the time lag between the pushing-out and the finger-opening movements becomes larger. As a result, more reliable object-placing can be achieved.

## Data Availability

The original contributions presented in the study are included in the article/Supplementary Material, further inquiries can be directed to the corresponding authors.
